# Association of virological breakthrough and clinical outcomes in entecavir-treated HBeAg-positive chronic hepatitis B

**DOI:** 10.1371/journal.pone.0221958

**Published:** 2019-08-30

**Authors:** Yi-Jie Huang, Sheng-Shun Yang, Hong-Zen Yeh, Chi-Sen Chang, Yen-Chun Peng

**Affiliations:** 1 Division of Gastroenterology, Department of Internal Medicine, Taichung Veterans General Hospital, Taichung, Taiwan; 2 School of Medicine, Chung Shan Medical University, Taichung, Taiwan; 3 School of Medicine, National Yang-Ming University, Taipei, Taiwan; 4 Department of Internal Medicine, Chiayi branch of Taichung Veterans General Hospital, Chiayi, Taiwan; National Taiwan University Hospital, TAIWAN

## Abstract

**Background & aims:**

To evaluate virological breakthrough (VBT) and the risk of hepatocellular carcinoma (HCC) in HBeAg-positive chronic hepatitis B (CHB) patients receiving entecavir (ETV) treatment.

**Methods:**

A retrospective cohort study was conducted in a tertiary referral hospital and a total of 228 HBeAg-positive CHB patients treated with ETV for more than 48 weeks were enrolled. Clinical outcome measures included HBeAg seroclearance, maintained virological response and the development of HCC.

**Results:**

During a median follow-up period of 197 weeks, VBT developed in 26 (11.4%) patients (VBT group), and the other 202 patients without VBT (non-VBT group). The overall cumulative rate of HBeAg seroclearance in the VBT group and non-VBT group were 23.1% and 23.8%, 27.1% and 37.9%, 27.1% and 55.1%, 27.1% and 74.1%, 27.1% and 76.7% from week 48 to 240, respectively(*p* = 0.013). The cumulative probability of maintained virological responses from week 48 to 240 were 7.69% and 21.78%, 7.69% in the VBT groups and 36.85%, 7.69% and 51.68%, 7.69% and 64.97%, 7.69% and 72.1% in the non-VBT groups, respectively (*p*<0.001). In the multivariate analysis, age (p<0.001) and virological response at week 24 (p = 0.005) were independently associated with VBT. Cox regression analysis showed that cirrhosis had carried the highest risk for HCC (HR = 4.99, CI = 1.14–21.81, p = 0.033). Subgroup survival analysis by Kaplan–Meier method showed that patients with VBT had higher incidence of developing HCC than without VBT in cirrhotic patients (50% (95%CI = 1–99%) vs 9% (95% CI = 1–9%); p = 0.048).

**Conclusions:**

VBT was associated with adverse clinical outcomes, including a low probability of HBeAg seroclearance, failure to achieve maintained virological responses, and a risk of developing HCC. Patients, particularly with cirrhosis, who had experienced VBT during ETV treatment, more likely developed HCC.

## Introduction

Hepatitis B virus(HBV) infection is a global health issue, which affects about 250 to 350 million people worldwide and is particularly prevalent in Asia and Africa. [1, 2]Chronic hepatitis B (CHB) infection is at increased risk of adverse liver outcomes including liver cirrhosis and hepatocellular carcinoma (HCC) over a period of years or even decades.[3, 4] In a 10-year follow-up study, up to 40% of CHB patients died due to liver cirrhosis and HCC.[5]

The interactions of host, viral, and environmental factors are a complex process, and have been documented to be associated with risks of cirrhosis and HCC. Serum levels of HBV DNA, HBeAg status, genotype, viral mutation and co-infection with other viruses are important factors for developing HCC.[6] High levels of HBV DNA are strongly associated with progression of cirrhosis and HCC development. [7–9]As such, HBV DNA viral suppression is an important target for reducing risks of subsequent progression of cirrhosis and HCC development, both of which improve survival rates. Therefore, CHB patients with HBeAg positivity are more likely to develop HCC, compared with those HBeAg-negative CHB patients.[10] Current guideline indicated that HBeAg seroconversion is an important milestone in treatment of HBeAg-positive CHB patients.[6, 11, 12]. Inactive HBV carriers still had a 5-fold higher risk for HCC development compared with HBsAg-negative subjects.[13]However, currently available agents like nucleotide/nucleoside analogue (NUC) can only inhibit viral replication and provide the HBeAg seroconversion in 40–50% of patients treated by entecavir (ETV) or tenofovir treated patients, but could not eradicate the HBV virus to achieve functional cure, as reported in a 5-year observational study.[11] Thus, patients with CHB typically require long-term NUC therapy due to the poor HBsAg seroclearance.[11, 14, 15]

Virological breakthrough (VBT) could be observed during long-term NUC therapy. CHB patients who experienced VBT have the risks of developing genotypic resistance and biochemical breakthrough[16]. Thus, ETV and tenofovir, with a high barrier to resistance, have currently replaced telbivudine, adefovir, and lamivudine (LAM) as the first-line oral agents for NUC-naïve patients. [6]

Recent studies showed that ETV is a high-potency antiviral agent for viral suppression. Resistance to ETV is rarely observed in long-term treatment-naïve patients.[17, 18] No information is currently available on the association between VBT and the risk of HCC. Here, we aimed to determine the factors and characteristics associated with VBT and the impact of VBT in ETV-treated HBeAg-positive CHB patients. We also assessed the clinical outcome and VBT in HBeAg-positive CHB patients treated with ETV.

## Materials and methods

### Patients

Under a protocol follow-up for CHB, we conduct a retrospective observational study of HBeAg-positive CHB patients who had received ETV treatment for at least one year between the period from August 2008 to October 2015 at a tertiary referral hospital. We enrolled a total of 228 consecutive ETV-treated HBeAg-positive CHB patients who were followed up every 12–24 weeks were enrolled in this study. Patients were excluded if they had a history of any of the following: (a) liver transplantation; (b) chemotherapy or immunosuppression agent; (c) combination therapy of NUCs and interferon (d) coinfection with hepatitis C virus, hepatitis D virus, or human immunodeficiency virus; (e) newly developed HCC within the first 12 months after initiating ETV to minimize the inclusion of pre-existing unidentified HCC; (f) HCC recurrence within the first 12 months after the last hospitalization for treatment of HCC after initiating ETV to minimize the inclusion of failure to response to previous therapy for HCC.

Of our patients, 28 were treatment experienced, and their characteristics, initial treatment regimen, and the clinical outcome based on virological response are listed in S1 Table.

### Clinical evaluation

Demographics and baseline characteristics of patients were regularly recorded. These items included liver cirrhosis, hepatitis B e antigen (HBeAg), HBV DNA, hepatic panel (albumin [Alb], alanine aminotransferase [ALT], total bilirubin (Bil-T), alkaline phosphatase [ALP]), prothrombin time (PT), and alpha fetoprotein (AFP). HBV DNA, HBeAg, and image studies including abdominal sonography, CT, or MRI were routinely assessed every 3–6 months. Liver cirrhosis was defined by the presence of atrophy or the nodular pattern of liver parenchyma with or without splenomegaly from the ultrasound examination or existence of esophageal varices or gastric varices observed during upper endoscopy.

### Ethics

The study was approved by the Institutional Review Board of our institution (VGHTC CE16037B).

Informed consent was waived owing to the retrospective nature of study.

### Definition of virological breakthrough

VBT is defined as any increase in serum HBV DNA by an amount >1 log_10_ from nadir or redetection of serum HBV DNA at levels 10-fold over the lower limit of detection of the viral load after HBV DNA becomes undetectable.

### Clinical outcome

The primary outcomes of this study were serological change and viral suppression. HBeAg seroclearance was defined as HBeAg loss with or without the formation of anti-HBe antibody (HBeAb). Virological response was defined as an undetectable serum HBV DNA level during ETV treatment.

Maintained virological response was defined as HBeAg loss and undetectable level in serum HBV DNA during ETV treatment.

### HCC assessment

The secondary endpoint of this study was the development of HCC, which was defined as newly-developed HCC or the recurrence of HCC. The HCC diagnosis was based on histology finding in liver biopsy or imaging, including CT or MRI accompanied by tumor marker according to practice guidelines[19].

### Laboratory methods

HBV DNA was determined by real-time PCR assay (Roche CobasTaqMan HBV Test). HBsAg and HBeAg were determined by electrochemiluminescence immunoassay (Roche Diagnostics, Mannheim, Germany).

### Statistical analysis

Statistical tests were done with the IBM SPSS Statistics package for Windows, version 22.0 (IBM Corp., Armonk, New York, USA. Categorical variables were compared with the Chi-squared test or Fisher’s exact test. Continuous variables were expressed as median and interquartile range, and compared by Mann–Whitney U test. The cumulative rates of HBeAg seroclearance, maintained virological response, and newly- developed HCC were calculated using the Kaplan–Meier method and compared with the log rank test. Cox proportional hazard model was used to analyze factors associated with VBT and newly- developed HCC, and significant factors in the univariate analysis were subjected to multivariate analysis to determine independent predictive factors. Statistical significance was set at p < 0.05.

## Results

### Comparison of patients’ characteristics based on development of VBT

Table 1 shows clinical characteristics of 228 ETV-treated HBeAg-positive CHB patients in relation to virological breakthrough. During a median follow-up period of 197 weeks, VBT was found in 26 of the 228 HBeAg-positive CHB patients. The VBT group was significantly older than the non-VBT group (median age 52.5(40–61) vs. 43.0 (34–52), *p* = 0.004). Of the 26 patients with VBT, three received other NUC therapy before receiving ETV monotherapy.

**Table 1:** Clinical characteristics of the VBT group and non-VBT groups before treatment.

	Total (n = 228)	Virologic breakthrough		*p* value
		Non (n = 202)	Yes (n = 26)	
Male	141 (61.8%)	123 (60.9%)	18 (69.2%)	0.542
Treatment Naïve	200 (87.7%)	177 (87.6%)	23 (88.5%)	1.000
Liver cirrhosis (n = 202 vs 25)	54 (23.8%)	47 (23.3%)	7 (28.0%)	0.783
DM^f^	23 (10.1%)	21 (10.4%)	2 (7.7%)	1.000
Fatty liver	91 (39.9%)	80 (39.6%)	11 (42.3%)	0.958
Age	44.0 (35–53)	43.0 (34–52)	52.5 (40–61)	0.004**
Cr (mg/dl) (n = 196 vs 23)	0.9 (1–1)	0.9 (1–1)	0.8 (1–1)	0.338
Glucose (mg/dl)(n = 87 vs13)	104 (90–123)	103 (90–122)	117 (98–137)	0.114
AFP (ng/ml) (n = 193 vs 25)	8.0 (5–16)	8.2 (5–16)	7.4 (6–14)	0.917
Albumin (g/dl) (n = 100 vs 14)	3.9 (4–4)	3.9 (4–4)	3.8 (3–4)	0.189
ALP (U/L) (n = 119 vs 17)	116.5 (90–162)	116.0 (89–156)	135.0 (92–182)	0.218
ALT(U/L) (n = 200 vs 25)	133.0 (73–311)	133.0 (72–321)	128.0 (73–281)	0.752
PLT (10^3^/CUMM) (n = 194 vs 25)	168 (119–225)	168.5 (122–225)	167 (89–228)	0.418
Bil-T(mg/dL) (n = 181 vs 22)	0.9 (1–2)	0.9 (1–1)	0.9 (1–2)	0.458
Follow-up time (week)	197.0 (140–289)	199.0 (141–296)	194.0 (127–249)	0.507
Pre-treatment HBVDNA (log10 IU/mL) (n = 199 vs 25)	7.3 (6–8)	7.3 (6–8)	6.6 (6–8)	0.979
PT (s) (n = 181 vs 22)	11.1 (11–12)	11.1 (11–12)	11.2 (10–13)	0.840

Chi-square test.

^f^ Fisher’s Exact Test. Mann-Whitney U test.

*P<0.05,

**P<0.01.

Continuous data were expressed median and IQR.

Categorical data were expressed number and percentage.

Abbreviations: VBT, Virologic breakthrough; DM, diabetes mellitus; Cr, creatinine; AFP, Alpha-Fetoprotein; ALP, Alkaline phosphatase; ALT, alanine aminotransferase; PLT, platelet counts; Bil-T, Bilirubin total; PT, prothrombin Time.

### Outcome of patients with VBT

**Fig 1:**
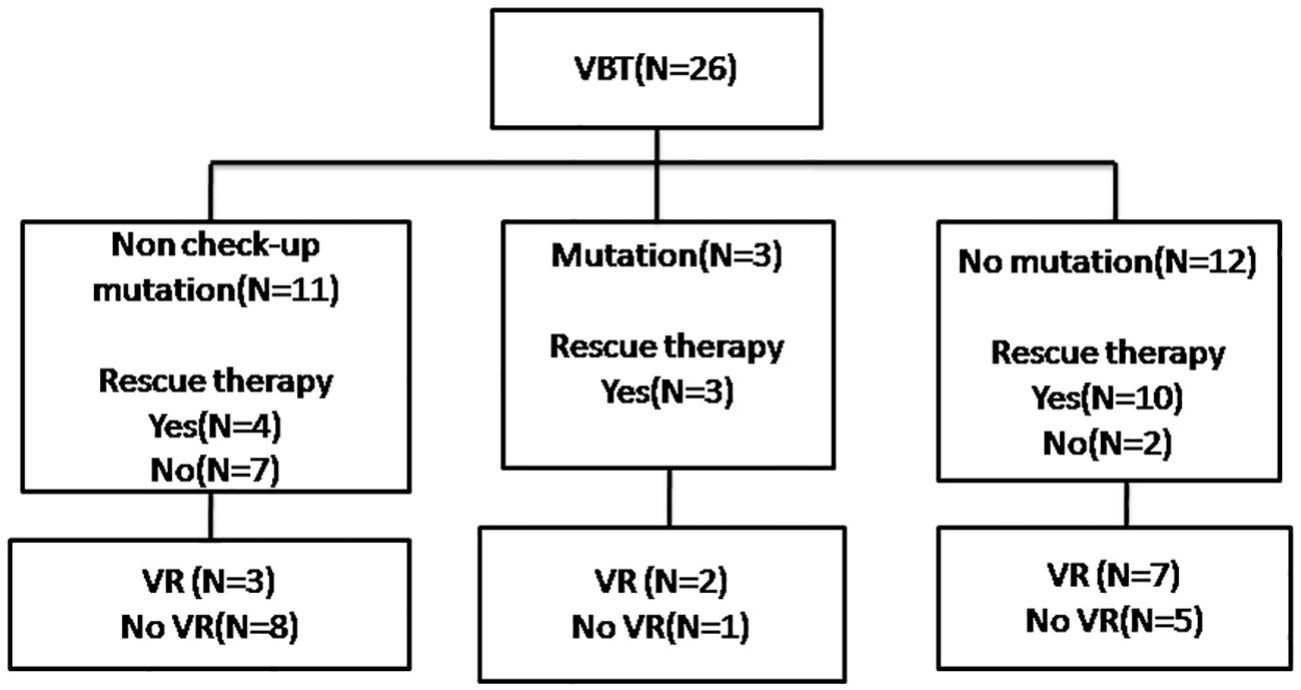
Outcomes of patients with VBT. VBT, virological breakthrough. VR, virological response.

Among VBT-experienced patients, three had antiviral drug resistance mutations; 17 started of rescue therapy including ETV plus adefovir combination or tenofovir monotherapy; 12 of them showed undetectable serum level of HBV DNA after the rescue therapy (Fig 1). Of the 26 patients with VBT, 6 died subsequently after the development of VBT and 4 had HBeAg seroclearance after receiving rescue therapy. The major cause of death included liver related multiple organ failure directly related to HCC (S2 Table). After development of VBT, six patients died due to the following two causes: liver failure (N = 2) or HCC (n = 4). As VBT was found at the early period of liver-related complication, so we had considered VBT was not the direct cause of death in these patients.

### Treatment response in relation to VBT

**Fig 2:**
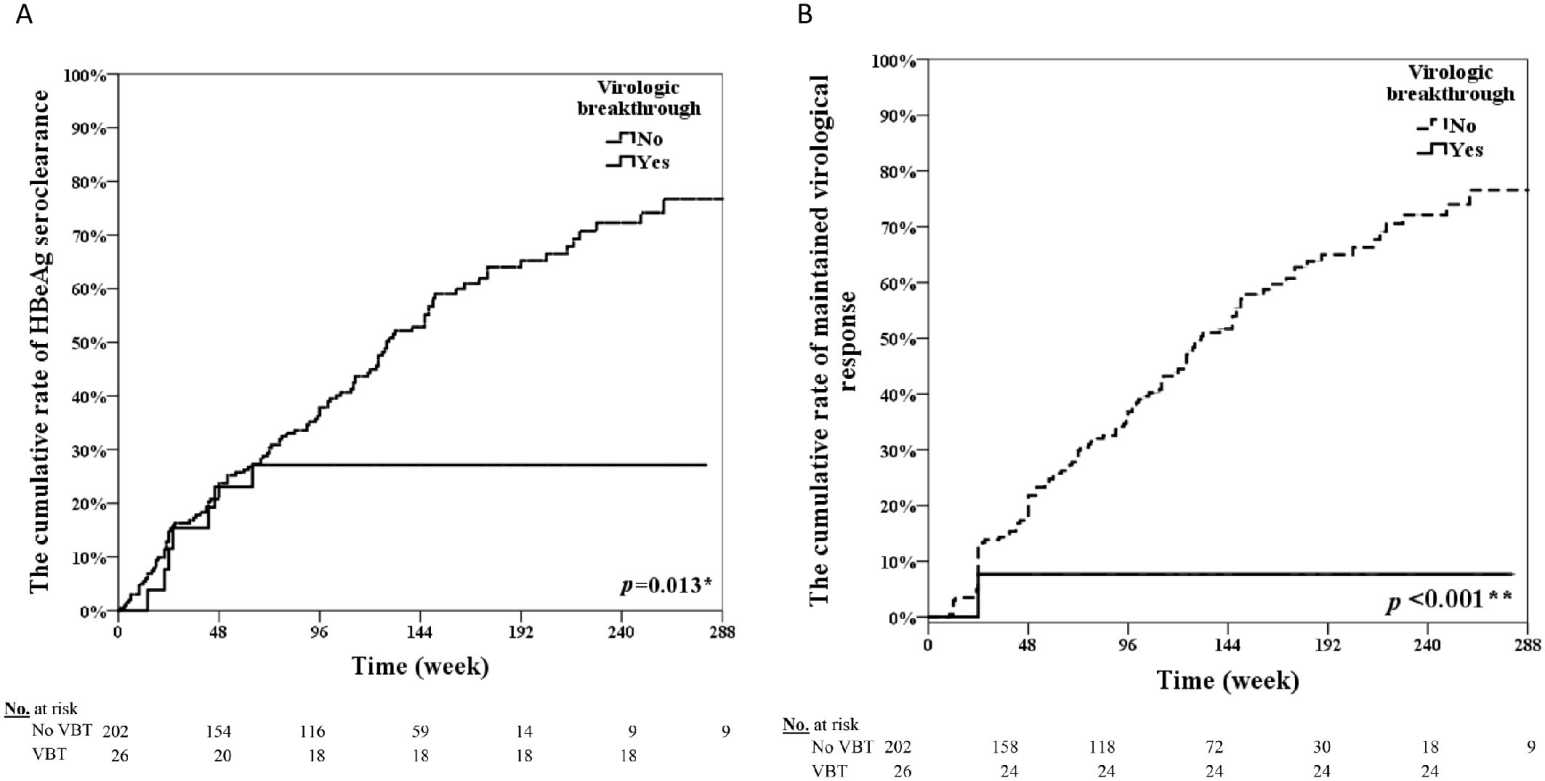
The impact of virological breakthrough in the clinical outcomes. (A)The overall cumulative rate of HBeAg seroclearance. (B)The cumulative rate of maintained virological response.

Fig 2A showed the cumulative rates of HBeAg seroclearance in the VBT group from weeks 48 to 240 as 23.1%, 27.1%, 27.1%, 27.1%, and 27.1%. These levels were significantly lower than those in the non-VBT group (23.8%, 37.9%, 55.1%, 74.1%, and 76.7% in the non-VBT group from weeks 48 to 240, *p* = 0.013). Fig 2B shows similarly the cumulative rates of maintained virological response in the VBT group; 7.67%, 7.67%, 7.67%, 7.67%, and 7.67%, which were significantly lower compared with those in the non-VBT group (21.78%, 36.85%, 51.68%, 64.97%, and 72.1% in the non-VBT group from weeks 48 to 240; *p*<0.001).

### Predictors for VBT

Table 2 shows the univariate analysis of risk predictors of VBT, including that the virological response at week 24 and age were two predictive factors in the univariate analysis. In the multivariable Cox regression analysis, age, and failure to achieve virological response at week 24 were also significant predictors of VBT (HR = 1.06, CI = 1.03–1.10, p<0.001; HR = 0.30, CI = 0.13–0.70, p = 0.005, respectively).

**Table 2:** Risk factor for virological breakthrough.

	Univariate analysis			Multivariate analysis		
	HR	95% CI	*p* value	HR	95% CI	*p* value
Age	1.04	(1.01–1.07)	0.004**	1.06	(1.03–1.10)	<0.001**
gender	1.38	(0.60–3.18)	0.446	2.23	(0.85–5.88)	0.104
naïve	1.27	(0.38–4.23)	0.698	1.02	(0.29–3.62)	0.980
Cr (mg/dl)	1.06	(0.82–1.37)	0.673	0.99	(0.74–1.33)	0.967
DM	0.67	(0.16–2.82)	0.580	0.59	(0.14–2.52)	0.472
VR24	0.33	(0.15–0.73)	0.006**	0.30	(0.13–0.70)	0.005*
ALT	1.00	(1.00–1.00)	0.557	1.00	(1.00–1.00)	0.588
Pre-treatment HBVDNA (log10 IU/mL)	0.95	(0.73–1.23)	0.695			

Cox regression.

*p<0.05,

**p<0.01.

Abbreviations: Cr, creatinine; DM, diabetes mellitus; VR24, virological response at week 24; ALT, alanine aminotransferase

### Comparison of patient’ characteristics based on development, or not of HCC

Clinical characteristics and demographics of the 228 ETV-treated HBeAg-positive CHB patients in relation to development of HCC are presented in Table 3. During a median follow-up period of 197 weeks, HCC development was diagnosed in 22 out of 228 HBeAg-positive CHB patients (newly developed HCC: n = 10, recurred HCC: n = 12). Those with HCC development including newly-developed HCC and recurred HCC were significantly older, with lower levels of platelet and ALT and had more conditions of liver cirrhosis and diabetes mellitus than those without HCC.

**Table 3:** Baseline clinical characteristics between non-HCC group newly diagnosed HCC group and recurrence of HCC group.

	Total (n = 228)		HCC						*p* value
			Non (n = 206)		New-diagnosed HCC (n = 10)		Recurrence of HCC (n = 12)		
Gender	141	(61.8%)	124	(60.2%)	9	(90.0%)	8	(66.7%)	0.156
Age	44.0	(35–53)	43.0	(33–52)	48.0	(43–61)	60.0	(51–66.8)	<0.001**
Treatment naïve	200	(87.7%)	181	(87.9%)	8	(80.0%)	11	(91.7%)	0.694
Fatty liver	91	(39.9%)	86	(41.7%)	3	(30.0%)	2	(16.7%)	0.182
Liver cirrhosis (n = 227)	54	(23.8%)	40	(19.4%)	6	(66.7%)	8	(66.7%)	<0.001**
DM	23	(10.1%)	15	(7.3%)	2	(20.0%)	6	(50.0%)	<0.001**
AFP (ng/ml) (n = 218)	8.0	(5–16.4)	8.0	(5–16.4)	9.7	(7–53.3)	7.7	(7–14.6)	0.441
Albumin (g/dl) (n = 114)	3.9	(4–4.3)	3.9	(4–4.3)	3.7	(3–4.3)	3.9	(3–4.2)	0.546
ALT(U/L) (n = 225)	133.0	(73–310.5)	139.0	(82–340.8)	69.0	(43–123)	48.0	(35–61.3)	<0.001**
Bil-T (mg/dl)(n = 203)	0.9	(1–1.5)	0.9	(1–1.5)	1.2	(0–1.4)	0.6	(0–1.1)	0.214
Body weight (kg) (n = 177)	65.0	(55–76.8)	65.0	(55–77)	66.4	(58–75.5)	64.0	(55–70)	0.970
Cr (mg/dl) (n = 219)	0.9	(1–1)	0.8	(1–1)	0.9	(1–1.1)	1.0	(1–1.4)	0.032*
Glucose (mg/dl) (n = 100)	104.0	(90–123)	101.0	(89–117)	125.5	(106–191.3)	127.0	(99–206)	0.007**
PLT (10^3^/CUMM) (n = 219)	168.0	(119–225)	176.0	(126–230.3)	101.0	(89–119)	121.0	(76–148.5)	<0.001**
Pre-treatment HBVDNA (log10 IU/mL) (n = 224)	7.3	(6–8)	7.4	(6–8)	6.1	(4–7.9)	6.4	(6–7.2)	0.074
PT (s) (n = 203)	11.1	(11–11.8)	11.2	(11–11.8)	11.1	(10–13)	11.2	(11–11.8)	0.966

Chi-square test. Kruskal Wallis test.

*p<0.05,

**p<0.01.

Continuous data were expressed median and IQR.

Categorical data were expressed number and percentage.

Abbreviations: HCC, hepatocellular carcinoma; DM, diabetes mellitus; AFP, Alpha-Fetoprotein; ALT, alanine aminotransferase; Bil-T, Bilirubin total; Cr, creatinine; PLT, platelet counts; PT, Prothrombin Time.

### Risk predictors for the development of HCC in patients with VBT

**Fig 3:**
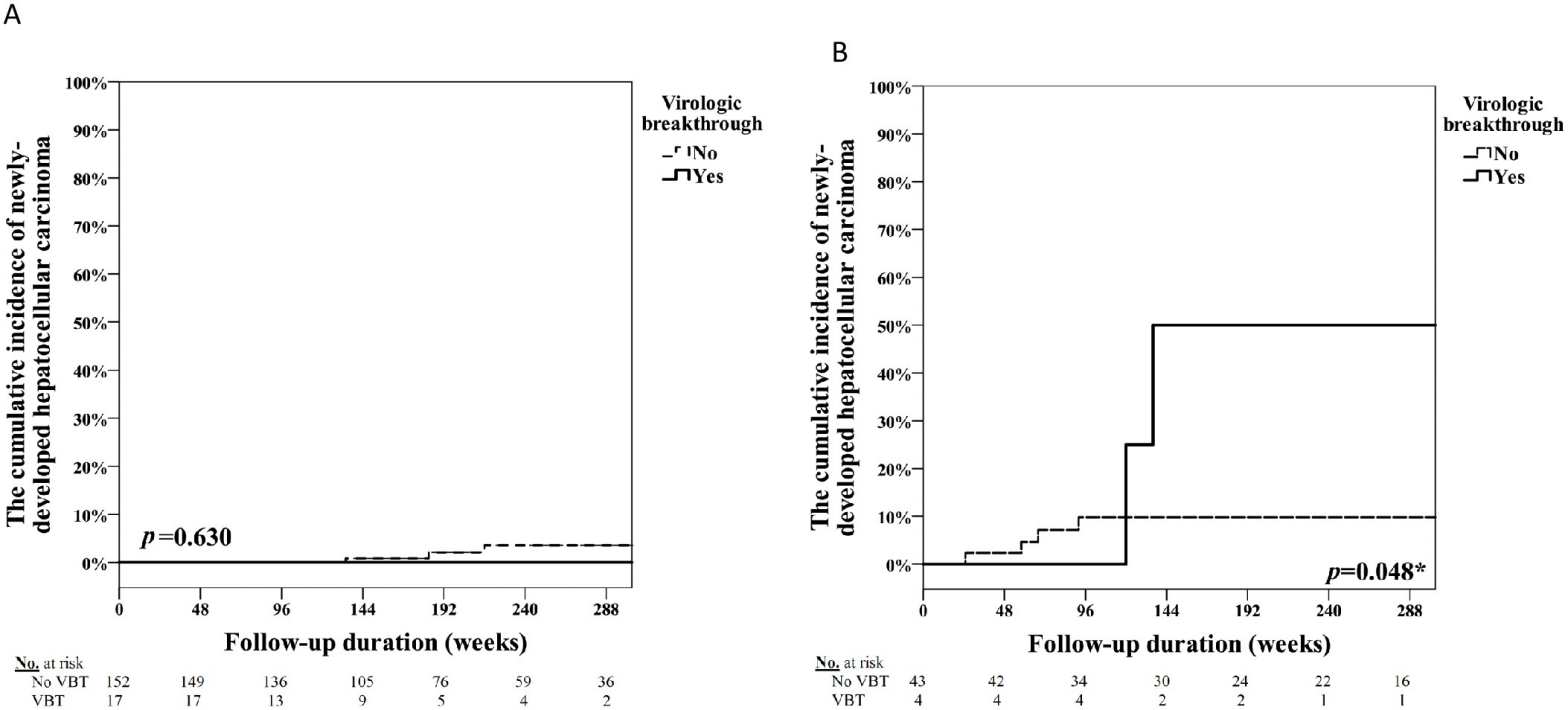
The impact of virological breakthrough in the development of newly-developed hepatocellular carcinoma. (A)The cumulative incidence of newly-developed hepatocellular carcinoma development in non-cirrhotic patients. (B)The cumulative incidence of newly-developed hepatocellular.

Table 4 shows the univariate and multivariate analyses on the association with newly-developed HCC. Univariate analysis showed that three factors: age, VBT, and liver cirrhosis were correlated with HCC development. Multivariate analysis identified liver cirrhosis as a significant risk predictor of HCC (HR = 4.99, CI = 1.14–21.81, p = 0.033). Fig 3 shows that VBT remained significant in cirrhosis subjects who developed new-diagnosed HCC but not in subjects without cirrhosis Subgroup survival analyses by Kaplan–Meier method showed that patients with VBT had higher incidence of new developing HCC than those without VBT in cirrhotic patients (50% (95%CI = 1–99%) vs 9% (95% CI = 1–9%); p = 0.048) during the follow up period of 240 weeks.

**Table 4:** Risk factors for newly-developed hepatocellular carcinoma.

	Univariate analysis			Multivariate analysis		
	HR	95% CI	*p* value	HR	95% CI	*p* value
Age	1.06	(1.01–1.11)	0.014*	1.04	(0.98–1.10)	0.165
Gender	5.17	(0.65–40.99)	0.120			
Treatment-naïve	0.84	(0.17–4.08)	0.827			
Cr (mg/dl)	0.95	(0.50–1.81)	0.885			
DM	2.61	(0.55–12.38)	0.226			
VBT	4.72	(1.22–18.36)	0.025*	3.12	(0.64–15.21)	0.159
Liver cirrhosis	7.17	(1.79–28.73)	0.005**	4.99	(1.14–21.81)	0.033*
VR24	0.88	(0.25–3.12)	0.843			
Pre-treatment HBVDNA (log10 IU/mL)	0.74	(0.53–1.03)	0.077			
HBV DNA>2000 at week 24	2.71	(0.58–12.78)	0.207			
ALT	1.00	(0.99–1.00)	0.232			

Cox regression.

*p<0.05,

**p<0.01.

Abbreviations: Cr, creatinine; DM, diabetes mellitus; VBT, virological breakthrough; VR24, virological response at week 24; ALT, alanine aminotransferase;

## Discussion

VBT was defined here as those associated with adverse clinical outcomes, including a low probability of HBeAg seroclearance, failure to achieve maintained virological response, and risk of HCC. We also found that age of patients and a failure to achieve virological response at week 24 were associated with the risk of VBT occurrence. The cirrhotic patients had markedly higher incidence of HCC, which is a finding of ours that is consistent with prior studies [20]. Therefore, VBT could be a risk factor for HCC development in cirrhotic patients.

The decline of HBV DNA at week 24 is important treatment response guiding further treatment. In this study, the relationship we found between BVT and residual HBV viremia at week 24 is consistent with previous reports. The 2-Year GLOBE trial showed that baseline HBV DNA and HBV DNA at week 24 were both risk factors associated with VBT in CHB patients receiving LAM and telbivudine[21]. Similarly, HBV DNA level at 6 months and 12 months are strongly associated with an increase in VBT among CHB patients under LAM or adefovir therapy[22–24]. Stefan et al. reported that non-detectable serum HBV DNA at week 24 is corelated with better outcomes, including HBeAg seroclearance and non-detectable HBV DNA at year 2 in CHB patients receiving telbivudine.[25] As previously reported, undetectable HBV DNA levels at week 24 is also a predictor of HBeAg seroclearance at 2 years of ETV therapy. [26, 27]To our best knowledge, data are yet reported regarding the relationship between VBT and ETV treatment. Our data showed that VBT was positively associated with poor subsequent therapeutic outcomes that include low rate of HBeAg seroclearance and failure to maintain viral suppression during ETV therapy. The clinical relevance of failure to achieve virological response at the early stage of NUC treatment is related to the high risk of developing VBT during follow-up.

Tomoo et al. reported a high and positive correlation between age and occurrence of VBT in CHB patients treated with LAM [28, 29]. Hashimoto Y et al. reported that young age protect against emergence of YMDD mutants over a 5-yearperiod of LAM therapy[30]. These results are similar to ours. However, the reason is not clear why in our study age is found to be an important factor in ETV-treated HBeAg-positive CHB patients with VBT. It may be use to explain why elderly patients have poor medical adherence or an inadequate immune response in inhibiting HBV replication.

The risk of HCC development is strongly correlated with serum HBV DNA levels.[9, 31] Thus, undetectable HBV DNA is an important goal in treating CHB patients under NUC therapy. Failures to suppress HBV viral load down to undetectable level during NUC therapy supposedly is an important risk factor for developing HC, especially cirrhotic patients[31, 32]. It was reported that annual incidence of HCC was 0.95% in LAM-treated CHB patients with sustained viral suppression, 2.18% in VBT and 5.26% in suboptimal response [33]. In addition, in an Asia population, the cumulative 5-year risk of HCC in LAM-resistance subjects is higher than those without resistance (p = 0.035).[34]However, antiviral drug resistance accounts for nearly 60% of VBT that is under long-term NUC treatment, and the cumulative rate of VBT at 5 years is 46.1%, confirmed VBT is 29.7%, and genotypic resistance at 5 years is 33.9%, respectively [35]. Our study showed that VBT is an independent risk factor for HCC in cirrhotic patients. As a consequence, suggest that CHB patients should receive high-potency NUC therapy to maintain viral suppression to reduce the risk of HCC in cirrhotic patients, and it is the important to monitor on-treatment HBV DNA regularly to detect occurrence of VBT. Once the occurrence of VBT is detected in cirrhosis patients, adequate surveillance testing should be performed for HCC at short intervals.

Fig 3B shows that VBT is an independent risk for developing HCC in cirrhotic patients. Nevertheless, the risk of HCC in non-VBT group is higher than VBT in the first 2.5 years with unclear mechanisms. In Table 1, patients with cirrhosis were not different in VBT group and non-VBT group. Cancer is a complex process related to host, virus or the interaction of both. Small case number may be also a factor weakened our conclusions. Givens larger case numbers, perhaps the borderline statistical significance we found currently, on cirrhosis an associated factor may improve.

Our strategies for rescue therapy, tenofovir monotherapy and ETV plus adefovir combination are rescue therapies. Supplementary Table 1 displays 5 patients who failed to achieve VR after receiving rescue therapy. Three patients died later due to liver failure and HCC. One patient was lost in follow-up and another receiving tenofovir still failed to achieve VR. Non-adherence could be a major cause of failure to VR. Improvement of medication adherence is crucial.

In our present study, we analyzed VBT and clinical outcomes in ETV treated HBeAg CHB patients. However, there are some limitations in our study. First, the number of cases was relatively small. Second, in this retrospective study, the presence of antiviral drug resistance mutations was not routinely checked at the baseline period and by the time when patients experienced VBT due to low HBV viral load. Third, because this was a retrospective study, drug adherence was not confirmed by reviewing medical records. Drug adherence has been shown to be an important factor in development of VBT. Fourth, there was no information on the quantitative HBsAg level in this study. Fifth, liver fibrosis and cirrhosis are important predictors for HCC development. As a retrospective study, we only collected baseline values of liver biochemistry tests. Liver fibrosis markers, such as Fib-4 value and APRI value were not used in this study. Besides, BMI were also were factors with clinical significance for HCC, due to incomplete dataset, we did not analyze BMI. Sixth, prior exposure to NUCs with low genetic barrier is associated with ETV resistance and VBT. Patients with prior exposures to NUC or having documented NUC resistance have increased risks at of developing ETV resistance under long-term ETV therapy. However, we only found three patients with antiviral drug resistance mutations in our study. Thus, we did not excluded patients with prior exposure to NUCs. Finally, HBV genotype was not determined in this study and therefore their effects on HCC development could not be assessed.

In conclusion, we found that in ETV treated HBeAg CHB patients, VBT was associated with adverse clinical outcomes. VBT also played a role in developing HCC in patients with cirrhosis. Future studies with more patients are needed for strengthening our clinical evidence.

## Supporting information

S1 Table Characteristics and clinical outcome of patients with treatment- experienced. (DOCX)

S2 Table Baseline characteristics and clinical outcome of patients experienced virological breakthrough. (DOCX)
