# Supplementary material for: Association of virological breakthrough and clinical outcomes in entecavir-treated HBeAg-positive chronic hepatitis B
Source: PLoS One. 2019 Aug 30;14(8):e0221958. doi: 10.1371/journal.pone.0221958 (PMC6716625; doi:10.1371/journal.pone.0221958)
Supplement: S1 Table — (DOCX) [file pone.0221958.s001.docx]

| Supplementary table 2 characteristics and clinical outcome of patients with treatment- experienced | | | | | | |
| --- | --- | --- | --- | --- | --- | --- |
| Patient No | Age | Gender | NUC-exposure | Mutation | Treatment | Clinical outcome-viroloigcal response |
| 1 | 67 | F | LAM | - | ETV | VBT |
| 2 | 51 | M | LAM | - | ETV | VR |
| 3 | 27 | M | LAM | + | ETV | VR |
| 4 | 45 | M | IFN | - | ETV | VR |
| 5 | 28 | M | LAM | - | ETV | VR |
| 6 | 54 | M | IFN | + | ETV→TDF | VBT |
| 7 | 51 | M | LAM | - | ETV | VR |
| 8 | 29 | M | IFN | - | ETB | VR |
| 9 | 49 | F | LAM | - | ETV | VR |
| 10 | 53 | M | LAM | - | ETV | VR |
| 11 | 38 | M | LAM | - | ETV | VR |
| 12 | 44 | F | LAM | - | ETV | VR |
| 13 | 30 | M | LAM | - | ETV | VR |
| 14 | 25 | M | IFN | - | ETV | VR |
| 15 | 50 | M | IFN&LAM | - | ETV | VR |
| 16 | 31 | M | LAM | - | ETV | VR |
| 17 | 26 | M | LAM ADF | - | ETV | VR |
| 18 | 18 | M | ADF | - | ETV | VR |
| 19 | 54 | M | IFN | - | ETV | VR |
| 20 | 35 | F | LdT | - | ETV | VR |
| 21 | 31 | M | LAM | - | ETV | VR |
| 22 | 47 | M | IFN | - | ETV | VR |
| 23 | 52 | M | LAM& ADF | - | ETV | VR |
| 24 | 48 | M | IFN | - | ETV | VR |
| 25 | 48 | M | LAM | - | ETV | VR |
| 26 | 61 | M | IFN | - | ETV | VR |
| 27 | 50 | M | LAM | + | ETV→TDF | VBT |
| 28 | 52 | M | LAM | - | ETV | VR |
| LAM, lamivudine ; ADF, adefovir ; TDF, Tenofovir ; ETV, entecavir; LdT, telbivudine ; VBT, virological breakthrough ; VR, virological response; IFN, interferon | | | | | | |
