# Supplementary material for: Association of virological breakthrough and clinical outcomes in entecavir-treated HBeAg-positive chronic hepatitis B
Source: PLoS One. 2019 Aug 30;14(8):e0221958. doi: 10.1371/journal.pone.0221958 (PMC6716625; doi:10.1371/journal.pone.0221958)
Supplement: S2 Table — (DOCX) [file pone.0221958.s002.docx]

| S2 table Baseline characteristics and clinical outcome of patients experienced virological breakthrough | | | | | | |
| --- | --- | --- | --- | --- | --- | --- |
| Pt No | Age | Sex | Mutation | Rescue therapy | HBV DNA (log10) | Clinical outcome |
| 1 | 69 | M | Non check-up | Add-on ADF | 2.1 | Achieved VR |
| 2 | 54 | F | ND | Add-on ADF | 2.3 | Achieved VR |
| 3* | 52 | M | L80I, A181, M204I | Switch to TDF | 6.1 | Achieved VR |
| 4 | 54 | M | L180M, M204V | Add-on ADF | 8.04 | No VR ;Death due to HCC and liver related MOF |
| 5* | 29 | M | YMDD,L528M | Switch to TDF | 6.82 | Achieved VR |
| 6 | 54 | M | ND | Switch to TDF | 5.6 | Achieved VR and HBeAg seroclearance |
| 7 | 47 | F | ND | Maintenance of ETV | 4.72 | No VR; Loss follow-up |
| 8 | 42 | F | ND | Switch to TDF | 7.56 | Achieved VR |
| 9 | 59 | F | ND | Switch to TDF | 4.44 | Achieved VR |
| 10 | 41 | M | ND | Add-on ADF | 8.2 | Achieved VR and HBeAg seroclearance |
| 11 | 35 | M | ND | Maintenance of ETV | 5.7 | No VR |
| 12 | 33 | M | Non check-up | Maintenance of ETV | 4.9 | No VR; Loss follow-up |
| 13 | 64 | M | ND | Switch to TDF | 5.04 | No VR and then switch to TDF ; still no VR |
| 14 | 73 | F | Non check-up | Maintenance of ETV | 2.59 | No VR; Death due to HCC and liver related MOF |
| 15 | 65 | M | ND | Add-on ADF | 2.48 | No VR ;Death due to HCC and liver related MOF |
| 16 | 60 | M | Non check-up | Add-on ADF | 2.15 | Achieved VR and HBeAgseroclearance |
| 17 | 42 | M | Non check-up | Maintenance of ETV | 6.57 | No VR |
| 18 | 56 | M | ND | Switch to TDF | 7.48 | No VR; Death due to liver related MOF |
| 19 | 38 | F | ND | Add-on ADF | 4.05 | Achieved VR and HBeAg seroclearance |
| 20 | 55 | M | Non check-up | Switch to TDF | 3.44 | Achieved VR and loss follow-up |
| 21* | 37 | M | ND | Add-on ADF | 4.61 | Non VR and then switch to TDF ; VR |
| 22* | 63 | M | Non check-up | Maintenance of ETV | 3.24 | No VR; Death due to liver related MOF |
| 23 | 61 | M | Non check-up | Maintenance of ETV | 3.00 | No VR |
| 24* | 69 | F | Non check-up | Maintenance of ETV | 2.14 | No VR; Death due to HCC and liver related MOF |
| 25 | 47 | M | Non check-up | Switch to TDF | 3.4 | No VR and loss follow-up |
| 26 | 39 | M | Non check-up | Maintenance of ETV | 3.76 | No VR and loss follow-up |
| a ND, mutation was not detected  b VR, virological response  *NUC-experienced  ADF, adefovir ; TDF, Tenofovi ; ETV, entecavir; VR, virological response; HCC, hepatocellular caricinoma; MOF, multiple organ failure. | | | | | | |
